# Treatment of reactive arthritis with biological agents: a review

**DOI:** 10.1042/BSR20191927

**Published:** 2020-02-20

**Authors:** Huiqiong Zeng, Baiwei Luo, Yue Zhang, Zhongyu Xie, Zhizhong Ye

**Affiliations:** 1 Shenzhen Futian hospital for Rheumatic diseases, Futian District, #22 Nonglin Road, Shenzhen 518040, Guangdong, China; 2 Department of Orthopedics, the Eighth Affiliated Hospital of Sun Yat-sen University, Futian District, #3025Shennanzhong Road, Shenzhen 518040, Guangdong, China

**Keywords:** biological agents, Cytokines, Reactive arthritis, review, treatment

## Abstract

The pathogenesis of reactive arthritis (ReA) has not been fully elucidated. In recent years, many researchers have confirmed that multiple cytokines are involved in the occurrence and development of ReA. Although ReA is self-limiting, it is still incurable for some patients who have no or a weak response to traditional drugs, such as non-steroidal anti-inflammatory agents, glucocorticoids and immunosuppressive agents. This is called refractory reactive arthritis. Currently, there is insufficient evidences for the treatment of refractory ReA with biological agents, though biological agents against cytokines have been developed over the past few years. This review summarizes the current development of clinical treatments of ReA with biological agents, which provides future investigations on refractory ReA with more evidence and references.

## Background

Reactive arthritis (ReA) is usually caused by the activation of T lymphocytes in joints triggered by genitourinary or gastrointestinal infection, releasing inflammatory mediators and leading to aseptic synovitis. At present, mechanisms of ReA pathogenesis are still unclear. ReA is generally regarded as having a close relationship with infection, genetic factors and immune abnormalities. One study has suggested that disordered gastrointestinal microflora might be related to the incidence of ReA [1]. ReA usually occurs in people aged 18–40 years, with no significant difference between males and females. Because of the inconsistency of diagnostic criteria, differences in clinical severity, a lack of specific biomarkers, and inheritance factors, the epidemiology of ReA, with an incidence of 0.6–30 per 10.000, varies around the world [2–5]. Although ReA is a self-limiting disease, as many as 63% of patients with ReA develop chronic arthritis and they often have no significant response to treatment with traditional drugs [6]. However, because of the high level of tumor necrosis factor (TNF)-α in ReA patients with chronic arthritis, the use of anti-TNF-α antibodies could improve the patients’ condition, suggesting that the occurrence and development of ReA are closely related to the levels of inflammatory cytokines [7,8]. Therefore, further studies need to focused on the development of new biological agents on treating ReA, which would be helpful for providing evidence and references.

## Pathogenesis of ReA

ReA is a pathological process in which environmental factors interact with genetic factors [9]. Pathogen infection plays a key role in ReA. Studies using polymerase chain reaction (PCR) and immunocytochemical staining have proven that definite bacterial triggers, such as lipopolysaccharides, or bacterial products can be found in the synovial tissue or fluid, and the persistence of these components is an important factor, resulting in ReA turning into chronic arthritis [10]. After local bacterial infection, the bacterial antigens or peptides are transported from the primary site into the synovial membrane by antigen-presenting cells (APC), leading to the activation of T-lymphocytes against bacterial antigens or peptides and the release of a large number of inflammatory cytokines, finally resulting in synovial inflammation. Chlamydia (*Chlamydia trachomatis*) infection is the most common factor causing ReA, which can persist in the host and cause typical reactive arthritis [2]. Approximately 5% of patients with acute *Chlamydia* infection will suffer from ReA [11]. When *Chlamydia* infects certain host cells, such as monocytes, the body releases pro-inflammatory cytokines, which induce persistent *Chlamydia* infection. This is mainly because *Chlamydia* in some way inhibits the combination of phagosomes and lysosomes, which makes *Chlamydia* live in cells; it is closely related to entering the stage of persistent infection [12,13]. Briefly, ReA development is dependent on live infection and it is correlated with cytokines, tissue damage and inflammation [14–16].

HLA-B27 plays an important supporting role in ReA and the most closely related one is the free heavy chain of HLA-B27 [17]. Studies have shown that HLA-B27 test results are positive in 50–80% of ReA patients [18]. However, HLA-B27 is not the only determinant of ReA. It has been proven that *HLA-B51, B60* and other genes may encode susceptibility to ReA. *HLA-B27* has multiple alleles that may affect the host response and disease susceptibility; among these, HLA-B*2703 increases the risk of the typical clinical triad of ReA [19]. Other data suggest that HLA-B27 may contribute to the persistence of bacteria in the host, especially *Chlamydia* and *Salmonella* [20,21]. So how does the susceptible *HLA-B27* gene participate in the occurrence and development of ReA? It was found that [17,22,23], (1) HLA-B27 folds more slowly than other types of HLA in endoplasmic reticulum assembly, which leads to the accumulation of the HLA-B27 homologous dimer and b2-microglobulin, as well as activating the inflammatory process; (2) the HLA-B27 heavy chain can activate natural killer cells, T-cells and B-cells, thus causing an inflammatory reaction; (3) microbial peptides mimic certain autopeptides, which increase the specificity of HLA-B27 and the reactivity of CD8+T lymphocytes, thus leading to autoimmune and inflammatory tissue damage.

**Figure 1:**
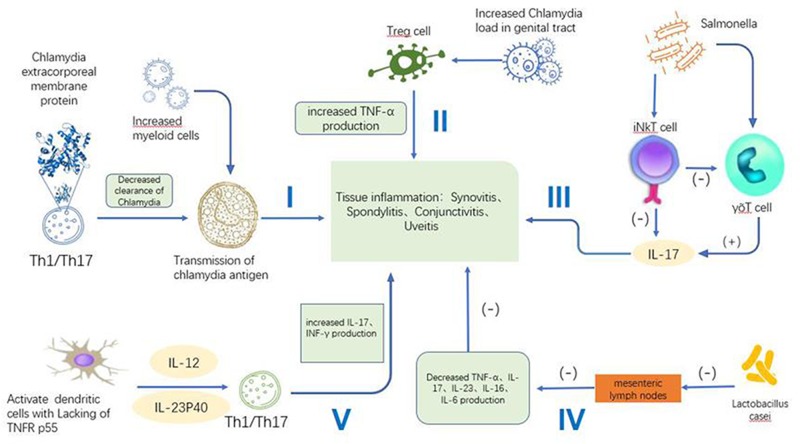
Five major functional pathways in pathogenesis of ReA I, The deceased ratio of Th1/Th17 and increased myeloid cells caused by *Chlamydia* inflammation lead to the transmission of *Chlamydia* antigen [27,28]. II, *Chlamydia*-induced reactive arthritis is TNF-dependent inflammatory disease. Increased TNF-α production and deficient Treg control promoted ReA [14]. III, iNKT cells play a protective role against Salmonella-induced ReA by down-regulating IL17-γδT cells [26]. IV, *Lactobacillus casei* can inhibit the expression of TNF-α, IL-17, IL-23, IL-1 and IL-6 in intestinal lymph nodes which lead to protective role in ReA. V, TNFRp55 regulates the cytokine production and enhanced the production of IFN-γ and IL-17, which developed severe chronic *Yersinia enterocolitica*-induced (ReA) [32–34]. **Note:** Th1/Th17:Helper T cell; iNKT cell: Constant-type natural killer cell; υδT cell: Innate immune cell; TNF-RP55: TNF receptor of p55; (-): inhibition; (+): activation.”

In addition to genetic factors, immune cells, such as dendritic cells and T-cells, also play important roles in ReA. In genetically sensitive individuals, abnormal physiological and pathological processes exist in the affected patient, including Th1 and Th17 cell differentiation, enhancement of the IL-17 response, the activation of IL-17-related T-cells and the release of various cytokines in intestinal lymph nodes. All these could promote the host’s immune response and the infiltration of immune cells (Figure 1). One study focused on whether there were cytokines in the joints of 11 ReA patients after infections with *Chlamydia, Pseudomonas aeruginosa* or *Salmonella*, and showed that under the stimulation of specific bacteria, mononuclear cells in the synovial fluid secrete a small amount of IFN-γ and TNF-α, while secreting a large amount of IL-10. After anti-IL-10 antibody was added or exogenous IL-12 was increased, the secretion of IFN-γ and TNF-α increased significantly in these cultures [24]. This proves that IL‐10–IL‐12 balance plays a key role in regulating the release of cytokines in the joints of patients with ReA. Meador et al. showed that TNF-α was involved in ReA, and an effective anti-TNF treatment also indirectly confirmed this point [25]. One study showed that iNKT cells, a unique subgroup of T lymphocytes, could provide protection from ReA induced by *Salmonella* through down-regulating the IL-17 produced by γδ T cells [26]. The level of IFN-γ was reduced in the peripheral blood of ReA patients [27]. The decreased ratio of Th1 to Th17 will lead to decreased clearance of *Chlamydia* [28]. Local migration of myeloid cells is activated by chlamydia pathogen-associated molecular patterns and transport of *Chlamydia* antigens spreads the inflammatory response from the genital tract to diseased tissue, and the myeloid antigen-presenting cells deliver *Chlamydia* antigens to antigen-specific TNF-producing T-cells locally. It is consistent with the detection of *Chlamydia* antigen-specific CD4+ and CD8+ T-cells in the joints of patients with *Chlamydia*-induced reactive arthritis [14]. In addition, myeloid cells are increased in infected SKG mice, predisposing them to higher levels of inflammation.

Another study has shown that IL-6 levels in the synovial fluid and plasma of patients with inflammatory arthritis was significantly higher than those in patients with osteoarthritis [29]. A study focusing on patients with Type S typhoid ReA showed that *Salmonella adventitia* proteins could stimulate the synovial immune cells to produce IL-17 or IL-23, which may be one of the causes of arthropathy [30]. Research has shown that *Lactobacillus casei* can inhibit the expression of TNF-α, IL-17, IL-23, IL-1 and IL-6 in intestinal lymph nodes, thus avoiding the activation of Th17 and Treg cells related to ReA pathogenesis; this may play a role in preventing ReA [31]. A study on SKG mice strongly supported the idea that the production of TNF responds to antigens from *Chlamydia* cells, leading to TNF-dependent inflammatory disease [14]. Either reducing *Chlamydia* burden through combined antibiotic therapy or reducing inflammation by TNF inhibitors reduced inflammation in SKG mice.

*Yersinia*-induced ReA progresses in mice lacking TNFR p55 [32]. *Yersinia enterocolitica* was confirmed as a pathogen of *Yersinia* and it has been shown that it can also produce *Yersinia pseudotuberculosis*-derived mitogen, a super antigen that is associated with ReA [33]. A later article [34] revealed the involvement of p40, a common subunit of heterodimeric IL-12 and IL-23, in increasing both IFN-γ and IL-17 production under TNFR p55 deficiency. Taken together, these data indicate that, in the absence of TNFR p55 signaling, Th1 and Th17 effector cells may have a critical function that sustains the inflammatory response in ReA processes.

This provides evidence for the treatment of human *Chlamydia*-induced ReA, indicating that a combination of antibiotics or TNF inhibitors can reduce the activity of ReA. The summary of ReA-related cytokines above provides a solid theoretical basis for the treatment of patients with biological agents and the development of new drugs by researchers.

## Biological agents for treating ReA

There is no magic bullet for ReA, especially refractory ReA. Clinicians often treat ReA patients on the basis of clinical experience [35]. Traditional drug treatment for ReA includes four categories of drugs: non-steroidal anti-inflammatory drugs, glucocorticoids, disease-modifying antirheumatic drugs and antibiotics. Of these, the effectiveness of antibiotic treatment is not sufficient. A meta-analysis showed that antibiotic treatment had no significant benefit for ReA [36]. However, a double-blind prospective study showed that combined antibiotic treatment was effective for *Chlamydia*-induced chronic ReA [37]. In clinical practice, it is common to encounter patients with ReA who do not respond well to traditional drugs. Therefore, clinicians have begun to hope for new biological agents for ReA. In recent literature on biological agents in the treatment of ReA, treatment with biological agents is mostly reported, but the literature has rarely been summarized [38–40]. There are no large-scale case–control trials using biological agents for the treatment of refractory ReA. Although most published studies are case reports and small-scale open clinical trials using biological agents for the treatment of refractory ReA, the therapeutic effect is exciting. Most patients with refractory ReA have significantly improved their symptoms and inflammatory markers of arthritis after using biological agents [38,39,41]. The pathogenesis of the disease and *in vitro* experiments support biological therapy. Most case reports confirm the safety and efficacy of the treatments. The relevant results are summarized below, and in Tables 1 and 2.

**Table 1:** Anti-TNF agents in ReA treatment

Author, Year (reference)	No. of patients	Anti-TNF agents	Dose (mg/kg)	Clinical infection	Microbial infection	Severe adverse reaction	Efficacy
Meyer, 2011 [35]	10	I, A, E	Not to mention	4wk<ReA	4wk<ReA	none	90%
Oili, 2003 [39]	2	I	3	Diarrhea	Yersinia	none	Yes
Gaylis, 2003 [40]	1	I	5	HIV	HIV	none	Yes
Gill, 2008 [41]	1	I	3	Urethritis	Chlamydia	none	Yes
Gaylis, 2012 [8]	1	I	3	HIV	HIV	none	Yes
Schafranski, 2010 [42]	1	I	5	Urethritis	Chlamydia	none	Yes
Wechalekar, 2010 [33]	1	I	6	Urethritis	Chlamydia	none	Yes
Flagg, 2005 [34]	10	E	25 mg/time	6wk<ReA	6wk<ReA	none	90%
Edrees, 2012 [43]	1	E	50 mg/ time	Urethritis	Chlamydia	none	Yes
Sánchez, 2007 [44]	1	A	40 mg/ time	pharyngitis	none	none	Yes
Ahogo, 2017 [38]	1	I	5	none	none	none	Yes

I: Infliximab, E: Etanercept, A: Adalimumab

HIV: Human Immunodeficiency Virus

**Table 2:** Other biologic agents (non TNF-α blockers) in ReA treatment

Author, Year (reference)	No. of patients	Anti-TNF agents	Dose (mg/kg)	Clinical infection	Microbial infection	Severe adverse reaction	Efficacy
Tanaka, 2009 [48]	1	Tozilizumab	8	Urethritis	none	none	Yes
Van, 2018 [54]	1	Secukinumab	300 mg/time	Not to mention	Not to mention	none	Yes

## Anti-TNF agents

Many studies have confirmed that the concentration of TNF-α in the synovial fluid of ReA patients increased significantly, causing obvious inflammation [7,25]. This laid a theoretical foundation for the treatment of ReA with anti-TNF antibodies. In fact, case reports and small open clinical trials have shown that anti-TNF antibodies were effective in most ReA patients without serious adverse effects

Meyer et al. studied 10 patients with refractory ReA who did not respond well to traditional drug therapy. All 10 patients were treated with anti-TNF antibodies, including five with infliximab, four with etanercept and one with adalimumab. IN nine cases, treatment was quick and effective, with CRP significantly decreasing and alleviated joint symptoms. One patient did not respond to infliximad. The specific reasons have not yet been discussed. The 10 cases were followed up for 6–50 months and no serious adverse events were found [40]. If there are ReA patients with tendinitis or finger or toe inflammation, and the effect of non-steroidal anti-inflammatory drugs and anti-rheumatic drugs to improve the condition is not obvious, then the anti-TNF antibody should be used as early as possible [42].

In 2017, Ahogo et al. reported that a 28-year-old man was diagnosed with ReA after unprotected sex. The patient presented with the typical conjunctivitis–urethritis–synovitis triad but was negative for HIV antibodies, syphilis, gonococcus and *Chlamydia*. Despite nonsteroidal anti-inflammatory drugs and glucocorticoid therapy, the patient’s condition deteriorated further, including severe weight loss, polyarthritis, keratotic plantar lesions, glans penis inflammation and marked elevation of CRP. Afterward, infliximab was administered at a dose of 5 mg/kg. This therapy had obvious effects on his arthritis and skin lesions, and the patient’s weight gradually recovered. The patient was followed up for 2 years. No serious AE or recurrence were found [43].

Oili et al. reported a middle-aged male patient with ankylosing spondylitis who developed multiple joint pain after a bout of diarrhea. Three classes of antibodies: IgM-, IgA- and IgG-class antibodies to *Yersinia enterocolitica* were detected 1 month later. ReA was diagnosed and treated with infliximab and improved markedly. At the same time, a healthy middle-aged man with upper respiratory tract infection developed knee arthritis and wrist arthritis 2 months later. His CRP level resolved significantly. The condition of the patient was alleviated after treatment with infliximab [44].

Gaylis et al. reported a middle-aged male patient with HIV-positive Wright’s syndrome who received infliximab (300 mg on weeks 0, 2 and 6), followed by antiretroviral therapy every 6–7 weeks. All his Wright’s syndrome symptoms were relieved after 6 months [45].

Gill et al. reported a young male patient with post-urethritis arthritis who had been treated with antibiotics, non-steroidal anti-inflammatory drugs, hormones and methotrexate for 3 months. Synovitis persisted and skin lesions worsened. A trial of infliximab was successful. Because TNF-α is an important component of the immune response, antagonizing TNF-α may increase the risk of infection, especially in HIV patients [46]. However, in a 10-year follow-up of an HIV-related ReA patient treated with infliximab and antiretrovirals, it was found that infliximab did not increase the risk of infection and did not affect antiretroviral therapy [8].

In 2010, Schafranski et al. reported a case of *Chlamydia*-related ReA treated with methotrexate and sulfasalazine. Infliximab was effective. However, the patient was not followed up to further monitor the occurrence of adverse events [47].

Wechalekar et al. reported that a young white woman who was HLA-B27-positive had severe knee swelling and pain caused by *Chlamydia* infection that could not be alleviated by routine treatment. She received 6 mg/kg TNF-α at 0, 2 and 6 weeks. Surprisingly, the patient’s symptoms were completely alleviated and no adverse events and arthritis recurrence occurred during the follow-up of 9 months [38].

Etanercept has been shown to reduce inflammation, improve symptoms and reduce joint damage in rheumatoid arthritis, and to be effective in the treatment of ReA and undifferentiated arthritis [25]. In a 6-month open clinical trial, 16 patients with ReA or undifferentiated arthritis were treated with etanercept [39]. Ten patients reached the end of the trial. The content of bacterial nucleic acid in synovial tissue before and after treatment was compared with that before treatment by PCR. At the same time, the therapeutic effect was evaluated by counting the involved joints and pain score. The results showed that treatment was effective in nine cases. After suffering severe side effects of infection, one case did not respond to treatment. In 2012, Edrees et al. reported that a case of ReA after *Chlamydia* infection, which was difficult to trait satisfactorily results with traditional medication, was successfully treated with etanercept [48].

Adalimumab has been reported to be effective in ReA after streptococcal infection, but more studies and clinical evidence are needed to further confirm its efficacy and safety [49].

## Interleukin-6 receptor antibody

Interleukin-6 receptor antibodies (such as tozilizumab) are widely used in RA, but they are rarely reported in ReA treatment. However, it has been reported that serum IL-6 levels in patients with ReA increased [50,51]. In addition, the concentration of IL-6 in the synovial fluid of patients with ReA was significantly higher than that of patients with rheumatoid arthritis, so it was proposed that the overproduction of IL-6 was related to the occurrence of ReA [28]. These findings support the application of interleukin-6 receptor antibodies in ReA to some extent. Tanaka et al. [52] reported a young woman with a 4-year history of ReA who had obvious joint symptoms and conjunctivitis, and was HLA-B27-positive. No bacteria were found in the synovial fluid culture before treatment, hormones and immunosuppressive agents were ineffective, but the symptoms of ReA improved rapidly and continuously after the use of tropizumab. This is the first case confirming the efficacy of tropizumab in treating ReA.

## Interleukin-17a monoclonal antibody

Since IL-17 has been found to play an important role in the pathogenesis of spondyloarthropathy [53], it has been shown that IL-17a monoclonal antibody is effective in the treatment of ankylosing spondylitis, psoriatic arthritis and rheumatoid arthritis without serious adverse events [54–56]. At present, there are only three IL-17a monoclonal antibodies on the market: secukinumab, ixekizumab and brodalumab. However, few trials and reports have been conducted in ReA. In one study, secukinumab was used to treat one case of active ReA. The clinical symptoms improved rapidly and no serious AE occurred in the 12-week study [57]. Obviously, more studies are needed to confirm the therapeutic effect of IL-17a monoclonal antibody on ReA.

## Summary and prospect

In reports of ReA treated with biological agents, no AEs such as severe infection, tumorigenesis or virus spread have been found. Most studies have shown good tolerance and the effect is still remarkable. Aph et al. showed that when TNF-α levels dropped, *Chlamydia* DNA levels in the synovium could be up-regulated. Therefore, although further research is needed, in patients with *Chlamydia*-induced ReA, this treatment may need to be treated with caution [13]. From ReA’s link to cytokines, we can see that there are still many biological agents that could be developed for cytokines or upstream regulatory factors, providing a new direction for clinical treatment of ReA. Hereby, we summarize key points:

- There is no magic bullet for reactive arthritis (ReA), especially refractory ReA. Clinicians are often left to treat ReA on the basis of clinical experience.
- ReA is a classic rheumatic disease, reflecting a dynamic interface between environmental triggers and genetic susceptibility. ReA development is dependent on infection history, and it correlates with cytokines, tissue damage and inflammation.
- The treatment of ReA with biological agents is occasionally reported, but has rarely been summarized.
- The pathogenesis of the disease and the experiments *in vitro* support the evidence of biological therapy for ReA; this article summarizes the safety and efficacy of the treatment.

## Abbreviations

PCR: polymerase chain reaction
ReA: reactive arthritis
TNF: tumor necrosis factor
